# The New Neurology

**Published:** 1899-01-21

**Authors:** 


### THE NEW NEUROLOGY.

In lecturing upon the New Neurology Sir William Gowers[1] shows how it explains certain facts which have been ascertained by clinical observations. But, first, as to what we mean by the new neurology. According to the older view the constituent elements of the nervous system, by branching and by union, formed one continuous complex network with paths for the nerve impulses due to union and continuity of the cell processes. The paths in actual use were determined not only by such union but also by differences in "resistance," this resistance, which permitted an energetic impulse to spread more widely than a slighter one, being itself varied in degree by functional activity, by repetition of the same kind of activity, and by other nerve influences from various sources. Thus nervous action might be "inhibited." So far as any attempt was made to localise the seat of this "resistance," it was thought of as lying in the cells or in the feltwork of uniting processes in the grey substance. Much of this conception of the structure and function of the nervous system is retained, but the more the methods of examination have been improved the less perceptible have become the actual unions between the branches of the cell-processes. Discontinuity has been found to be the rule, so that we must now look upon the nervous system as consisting of discontinuous elements, each a cell body with its processes, long and short, elements for which the word "neuron" has been all but universally adopted. The neuron, however, is not a homogeneous mass; it consists of a number of fibrils which not only occupy the "axons" or long processes, and the "dendrons" or short processes, which pass into the grey matter, but also pass right through the mass of the nerve cell without interruption. What, then, of this nerve cell? This is no longer regarded as the seat of nerve impulses, or as the originator of nerve force. It is now known that on the nerve cell depends the life of the nerve fibres, both the axon and the dendrons, so that any part of the neuron separated from the cell immediately degenerates. Many facts previously obscure become more intelligible in the light of the new discoveries. The knowledge that the cell governs the nutrition of the fibre, and the belief that from it proceeded the nerve impulses, involved a correspondence in the direction of nutritional influence and of conduction, and it was found, in fact, that conduction and degeneration were in the same direction as a rule, but only "as a rule." It was untrue of the sensory nerves. These, coming from the cells of the ganglia on the posterior spinal roots, degenerate downwards, but they conduct upwards—an anomaly which has had to be simply ignored. The fact is now, however, seen to be in harmony with others. The arrest of the secondary degeneration in the grey matter, the fact that the degeneration of the fibre which enters the grey matter never passes on to the next nerve cell through which conducted impulses must pass, was mysterious when they were believed to be in continuity. The discontinuity explains it at once. The degeneration is limited to the single neuron. Again, the division of the axis cylinder at its terminal ramification was scarcely intelligible when the axis cylinder was regarded as a single conducting path from a single impulse-giving centre, the nerve cell; but the difficulty is cleared up when we see that the apparent division is simply the separation of its constituent fibrils, each a distinct conducting path.

[1] Lancet, Jan. 14.

